# Resistant Hypertension From Renal Artery Stenosis Leading to Heart Failure With Preserved Ejection Fraction

**DOI:** 10.1177/2324709618816501

**Published:** 2018-12-07

**Authors:** Motasem Alyamani, Jissy Thomas, Miriam Shanks, Gavin Y. Oudit

**Affiliations:** 1 University of Alberta, Edmonton, Alberta, Canada

**Keywords:** heart failure with preserved ejection fraction, renal artery stenosis, renal artery revascularization, resistant hypertension

## Abstract

Resistant hypertension remains an important cause of heart failure. In this article, we describe a case of resistant hypertension in a 63-year-old woman leading to heart failure and marked morbidity. Her clinical course was characterized by chronic pleural effusions and recurrent hospitalizations with respiratory failure and flash pulmonary edema associated with heart failure with preserved ejection fraction. Her transthoracic echocardiogram showed severe concentric left hypertrophy and diastolic dysfunction. The clinical phenotype was secondary to resistant hypertension due to bilateral renal artery stenosis, and her blood pressure and heart failure resolved after successful renal artery angioplasty. This case demonstrates how heart failure with preserved ejection fraction due to renal artery stenosis can easily go unrecognized especially in patients with multiple comorbidities. The potentially curable nature of this condition clearly warrants consideration especially in patients with multiple risk factors for atherosclerotic vascular disease.

## Case Report

**Figure 1.**
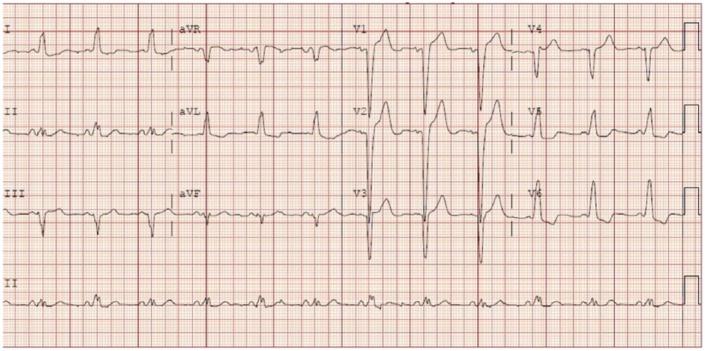
12-lead electrocardiogram showing sinus rhythm with a left bundle branch block.

A 63-year-old female with chronic bilateral pleural effusions and small pericardial effusion was transferred to the Mazankowski Alberta Heart Institute in October 2017 with respiratory failure secondary to flash pulmonary edema. She was intubated shortly after arrival and responded to diuresis with the resolution of her pulmonary edema. She was extubated after 2 days and had unchanged small pleural effusions. Her electrocardiogram on presentation (Figure 1) showed sinus rhythm with a left bundle branch block that was unchanged from her old electrocardiograms. Her transthoracic echocardiogram showed a normal ejection fraction and severe concentric left ventricular hypertrophy with diastolic dysfunction. Diastolic function was assessed using the American Society of Echocardiography guidelines.^1^

Her past medical history was significant for multiple hospital admissions with flash pulmonary edema and chronic pleural effusions since December 2016. Her previous investigations included a pleural biopsy that did not show any pathology, and following an episode of acute kidney injury, she underwent a renal biopsy, which was inconclusive. She also had a whole-body positron emission tomography scan that did not show any evidence of malignancy. She was known to have hypertension, chronic kidney disease, mild chronic obstructive pulmonary disease, schizoaffective disorder, and mild cognitive impairment. Her social history was significant for active smoking but no alcohol or illicit drug use. She was worked up for a possible inflammatory condition to explain her chronic pleural effusions. Her serum ANA (antinuclear antibody), anti-dsDNA, rheumatoid factor, erythrocyte sedimentation rate, and C-reactive protein were all negative. She had a positive pANCA and was referred to the rheumatology consult team. Her positive pANCA was felt to be nonspecific for any rheumatologic condition. She was also seen by the neurology team for a possible neurodegenerative disorder causing recurrent aspirations given her recurrent hospitalizations with respiratory failure. However, her swallowing assessment was completely normal, and she only had mild cognitive impairment on formal cognitive testing.

**Figure 2.**
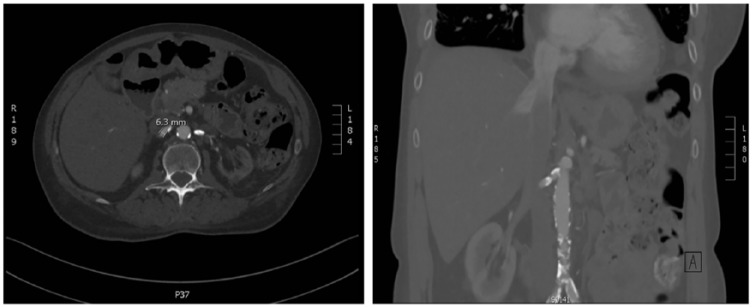
Horizontal and coronal views of abdominal computed tomography angiogram demonstrating bilateral atherosclerotic renal artery stenosis.

**Figure 3.**
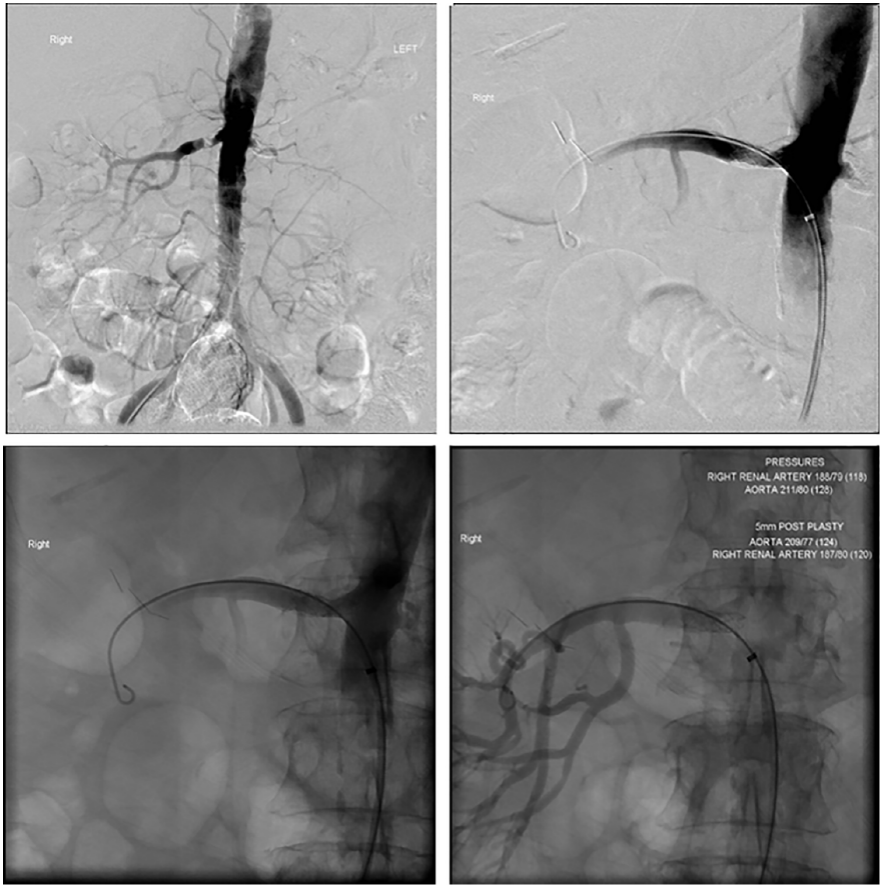
Pre and post right renal artery stenting on renal artery angiogram showing a clear demonstration of the restoration of renal arterial perfusion.

During her hospital stay, she was noted to have elevated systolic blood pressure above 190 mm Hg with diastolic blood pressure in 100 to 110 mm Hg range despite being on maximum doses of 5 antihypertensive medications (hydrochlorothiazide, bisoprolol, amlodipine, spironolactone, and terazosin). She developed anuric acute kidney injury shortly after starting the ACE inhibitor, ramipril. This was reversible with stopping the new medication, and her kidney function returned to baseline (creatinine level of 120 µmol/L) but her blood pressure was still elevated. At that point, bilateral renal artery stenosis (RAS) was suspected. A computed tomography angiogram of the renal arteries confirmed the diagnosis of atherosclerotic bilateral RAS (Figure 2). She then underwent a renal angiogram with right renal artery angioplasty and stenting (Figure 3). The left RAS was believed to be chronic as the left kidney was already atrophied. Her invasive systolic blood pressure was confirmed to be markedly elevated at 210 mm Hg. She tolerated the procedure well, and on the following day, her systolic blood pressure decreased to 120 mm Hg, and she felt lightheaded. Her antihypertensive medications were held and blood pressure was closely monitored. She was then introduced to a small dose (12.5 mg) of spironolactone for the mineralocorticoid blocking effect and 2.5 mg of bisoprolol to avoid β-blocker withdrawal. She tolerated both medications very well, and her blood pressure was in the normal range. She was also started on enteric-coated aspirin 81 mg daily and atorvastatin 80 mg daily for treatment of peripheral atherosclerotic disease.

She was followed closely as an outpatient and had no recurrent pulmonary edema. Her follow-up echocardiogram 8 months later showed normalization of the left ventricular mass, and reduction in the left atrial size and right ventricular systolic pressure (Table 1; Video 1, available in the online version of the article.).

**Table 1.** Key Echocardiographic Data Showing a Marked Reversal of Left Ventricular Hypertrophy and Left Atrial Size Following Renal Arterial Stenting.

	Prior to Renal Artery Stenting	Post Renal Artery Stenting
LV mass	138 g/m^2^	77 g/m^2^
RWT	0.8	0.5
LA volume index	33 mL/m^2^	21 mL/m^2^
MV E/A	0.72	0.73
E/e′	20.3	14.4
TR max PG	38 mm Hg	15 mm Hg

Abbreviations: LV, left ventricle; RWT, relative wall thickness; LA, left atrium; MV E/A, mitral valve peak velocity of early filling to peak velocity of late filling ratio; E/e′, mitral peak velocity of early filling (E) to early diastolic mitral annular velocity (e′) ratio; TR max PG, Bernoulli equation derived pressure gradient from the peak tricuspid regurgitation velocity.

In summary, a unifying diagnosis that explained her chronic pleural effusions and recurrent hospitalizations with respiratory failure (likely due to flash pulmonary edema) was heart failure with preserved ejection fraction (HFpEF) secondary to resistant hypertension due to bilateral RAS. This case demonstrates how HFpEF due to RAS (a potentially curable condition) can easily go unrecognized especially in patients with multiple comorbidities if a high index of suspicion is not maintained.

## Discussion

HFpEF is defined as signs and symptoms of heart failure in the presence of normal ejection fraction (>50%) and diastolic dysfunction.^2,3^ Although the presence of diastolic dysfunction alone on echocardiogram is not enough to make the diagnosis of HFpEF, it is an independent risk factor for heart failure hospitalization and death.^4^ HFpEF compromises at least one third to half of heart failure cases,^4,5^ is more prevalent in women, and is often associated with hypertension.^2,5^ It is not uncommon for HFpEF to go unrecognized especially in patients with multiple comorbidities in part due to the lack of a gold standard test to diagnose it. Our patient’s chronic pleural effusions were only part of her whole clinical picture and drove clinical investigations away from the underlying diagnosis. She underwent numerous invasive investigations including a pleural biopsy, a kidney biopsy, and a whole-body positron emission tomography scan.

Renovascular hypertension is a common correctable cause of secondary hypertension accounting for approximately 30% of cases.^6^ Atherosclerotic RAS should be suspected in all patients with resistant hypertension over the age of 55 years. Recurrent flash pulmonary edema and bilateral RAS is labelled as a distinct entity, the Pickering Syndrome, after the author who first described it in 11 hypertensive patients.^7^ Severe diastolic dysfunction and elevated LVEDP (left ventricular end-diastolic pressure) are the main pathophysiologic mechanisms to explain this phenomenon.

Another important clue to bilateral RAS is kidney dysfunction with the institution of ACE inhibitor therapy. Angiotensin II helps maintain or raise the intraglomerular pressure by preferentially vasoconstricting efferent glomerular arterioles resulting in glomerular filtration rate (GFR) autoregulation.^8^ By blocking the renin-angiotensin system in renovascular hypertension, this mechanism is disturbed and a reduction in GFR occurs. This is more pronounced in bilateral RAS as opposed to unilateral where the normal kidney compensates by increasing GFR. Many patients, however, with both unilateral and bilateral RAS, tolerate ACE inhibitors.^9^ In fact, Chrysochou et al^9^ showed that reducing intraglomerular pressure with renin-angiotensin blockade has a protective effect on renal function and a mortality benefit in these patients.^10^ This is thought to be through the same mechanism that ACE inhibitors delay the progression of other forms of chronic kidney disease, that is, reducing proteinuria and the vascular protective effect on diseases that usually accompany RAS like coronary artery disease.

Our patient had a more classic response to ACE inhibitor resulting in acute renal failure with anuria. This may be related to the fact that her left kidney was atrophied and possibly nonfunctional and her right renal artery was severely stenosed, and the only way for her to have a reasonable GFR was by maintaining a systolic blood pressure above 190 mm Hg. While no randomized controlled trials have examined the clinical benefits of renal artery revascularization, a small number of small retrospective observational studies demonstrated significant benefit in heart failure and blood pressure control and delaying progression of kidney dysfunction in patients with renal artery sentosis.^10^ Other studies observed mortality benefit with renal artery revascularization in patients with recurrent flash pulmonary edema and heart failure.^11,12^ Kawarada et al^12^ were able to demonstrate an effect of improved left ventricular filling pressure and pulmonary artery pressure determined by echocardiography in a case of renovascular hypertension after revascularization.^12^

Revascularization can also enable treatment with renin-angiotensin blockers in patients with RAS who may derive mortality benefit from these medications if they are tolerated.^9^ Our patient had a rapid improvement in her blood pressure control within 24 hours after her procedure, and it has remained controlled in follow-up. She has had no further admissions with heart failure and is currently functioning at New York Heart Association class I.

In conclusion, bilateral RAS causing resistant hypertension is an important and prevalent cause of HFpEF. A high index of suspicion is required to diagnose this syndrome, especially in elderly patients with other forms of atherosclerosis. Randomized controlled trials will be difficult to conduct in this patient population given the small numbers, but if ever done, we expect them to better demonstrate benefits of revascularization, especially in the sicker patients with recurrent flash pulmonary edema.

## Supplementary Material

Supplementary material

Supplementary material
